# Nanofibrillar Peptide Hydrogels for the Immobilization of Biocatalysts for Chemical Transformations

**DOI:** 10.1002/marc.201400027

**Published:** 2014-03-07

**Authors:** Christopher Hickling, Helen S Toogood, Alberto Saiani, Nigel S Scrutton, Aline F Miller

**Affiliations:** School of Chemical Engineering and Analytical Science, Manchester Institute of Biotechnology, University of Manchester131 Princess Street, Manchester, M1, 7DN, UK; Manchester Institute of Biotechnology, Faculty of Life Sciences131 Princess Street, Manchester, M1, 7DN, UK; Manchester Institute of Biotechnology, School of Materials, University of ManchesterManchester, M1, 3 9PL, UK

**Keywords:** biocatalysis, chemical transformation, peptide hydrogel, self-assembly

## Abstract

Enzymes are attractive, “green” alternatives to chemical catalysts within the industrial sector, but their robustness to environmental conditions needs optimizing. Here, an enzyme is tagged chemically and recombinantly with a self-assembling peptide that allows the conjugate to spontaneously assemble with pure peptide to form β-sheet-rich nanofibers decorated with tethered enzyme. Above a critical concentration, these fibers entangle and form a 3D hydrogel. The immobilized enzyme catalyzes chemical transformations and critically its stability is increased significantly where it retains activity after exposure to high temperatures (90 °C) and long storage times (up to 12 months).

## 1. Introduction

Enzymes have great potential as biocatalysts for the industrial manufacture of fine chemicals and pharmaceutical intermediates, and as such have been the topic of increasing academic and industrial attention over recent years.[1,2] This is primarily due to the number of advantages offered by enzymes in comparison to traditional chemical catalysts. For example, they often have very high chemo-, regio-, and enantioselectivity, are environmentally acceptable, operate under relatively mild reaction conditions, and minimize undesired side reactions.[3,4] However, turning attractive laboratory-scale biocatalysis into a viable manufacturing process remains a major obstacle for industry as natural enzymes rarely contain all the properties required for cost-effective industrial-scale chemical production. For example, enzymes may lack sufficient stability under industrial reaction conditions, compromising biocatalyst reuse and limiting productivity.[5,6] One strategy to increase biocatalyst stability is to immobilize the enzymes onto a surface.[7] In this communication, we demonstrate a method to do this that leads to increased longevity and stability of the enzyme in addition to its ability to perform a biotransformation reaction that provides near optical purity of products and high product yields.

**Scheme 1:**
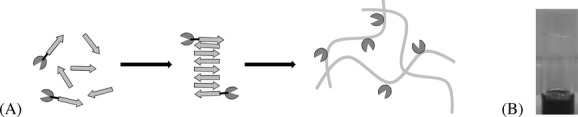
A) Diagrammatic representation of the combined self-assembly of VKVKVEVK–PETNR conjugates with pure VKVKVEVK peptide, where the peptide component of the conjugate contributes to fiber self-assembly, thus immobilizing the enzyme onto the fiber surface. B) Photograph of a 40 mg mL^−1^ self-assembling VKVKVEVK hydrogel containing 84 × 10^−6^ m of SPep–PETNR.

Methods to immobilize enzymes currently in the literature typically involve i) physically adsorbing the enzyme onto/within an inert, insoluble matrix such as calcium alginate; ii) physically entrapping the enzyme within a micellar structure, or iii) adsorbing it onto a membrane surface.[8–13] Physical adsorption and entrapment techniques are limited to very specific working conditions, as enzyme desorption may occur with only slight changes in temperature, ionic strength, or hydrogen ion concentration.[14] Other reports of physical adsorption outlined the physical entrapment of enzymes within Fmoc-dipeptide hydrogels, and reported that this increased the rate of bioconversion in organic co-solvents versus aqueous reactions.[15] Such systems have also physically entrapped enzymes for biosensing applications.[16] In these papers, the enzyme was noncovalently entrapped within the hydrogel matrix, and hence rapidly leached into the reaction media during the biotransformation. Consequently, this minimized the longevity and efficacy of this immobilization technique. Nevertheless, molecular hydrogels are ideal natural materials for the support of enzymes, as they reversibly self-assemble under aqueous conditions at physiological temperatures.[17] The amphiphilic nature of the peptide nanofibers formed would also facilitate the transportation of the substrate and product through the hydrogel before and after catalytic transformation, as well as cope with the presence of a co-solvent. To avoid enzyme leaching from the hydrogels, we decided to covalently attach an enzyme to a β-sheet self-assembling peptide and dope a small percentage of this conjugate into a pure peptide matrix. It was proposed that the peptide component from the conjugate would self-assemble within the pure β-sheet peptide structure, and hence immobilize the enzyme on the surface of the fiber. A schematic of the proposed self-assembly is given in Scheme 1. An advantage of this approach is that the incorporation of the enzyme is controlled simply via physical mixing, and has the potential to incorporate multiple enzymes within the one hydrogel. Moreover, many design rules are now emerging to predictably control the structure and properties of such β-sheet peptide hydrogels simply by changing the chemistry and concentration of the molecular building block and the biological microenvironment (e.g., ionic strength, temperature, and co-solvent).[17–22] This in turn permits the tailoring of the microenvironment to suit different enzymes (pH, ionic strength), provides control over the substrate and product diffusion rate, and allows a broadening of the scope of enzymes towards water-insoluble substrates via the use of co-solvents.

Here, we outline two synthesis strategies explored for the preparation of peptide–enzyme conjugates; chemical coupling (SPep–PETNR) of the peptide to the enzyme via click chemistry,[24–27] and genetic expression (CPep–PETNR) of the full peptide–enzyme conjugate. We go on to describe the physical properties of the matrices formed when mixing either SPep–PETNR or CPep–PETNR conjugate with pure peptide, and demonstrate that in both cases the enzyme remains active. Moreover, we show that these biocatalysts immobilized within our hydrogels show enhanced stability and longevity, in comparison to enzyme that is simply physically mixed within the hydrogel and or free in solution.

## 2. Experimental Section

### 2.1. Materials and Equipment

All reagents were of analytical grade. All medium components were obtained from Formedium, with all other chemicals were obtained from Sigma-Aldrich.

### 2.2. Hydrogel Formation

Hydrogels were formed by dissolving 40 mg of VKVKVEVK in 400 μL HPLC-grade water, adjusting to pH 7.0 ± 0.1 using 0.5 m NaOH and 0.5 m HCl. For hydrogels doped with free or conjugated enzyme, 3.4 mg of pentaerythritol tetranitrate reductase (PETNR) (in 50 × 10^−3^ m TRIS pH 7.0) post pH adjustment was added to the peptide solution, and HPLC-grade water added to give final peptide and PETNR concentrations of 40 mg mL^−1^ and 84 × 10^−6^ m, respectively. The standard tilt-test was used[17,28–30] as a crude test for hydrogelation, where a gel was defined when the sample did not flow upon inversion of the vial. For long-term stability studies, hydrogels were simply stored in the fridge for various periods of time up to 12 months and simply removed and allowed to equilibrate at 37 °C before subsequent rheology and activity measurements.

### 2.3. Oscillatory Rheology

The mechanical properties of each sample were obtained using a TA instruments AR-G2 rheometer equipped with a Peltier stage. Parallel plate geometry with a 250 μm gap was used with a solvent trap to minimize evaporation. Strain amplitude sweeps were performed to identify the linear viscoelastic region (LVR). The elastic (*G*′) and viscous (*G*′′) moduli of the hydrogels were recorded as a function of radial frequency between 0.1 and 100 rad s^−1^ at 0.3% strain. All measurements were performed at least three times.

### 2.4. PETNR Release from Hydrogels

1 mL of PBS buffer pH 7.0 was placed on the top of each hydrogel sample, to form a buffer layer into which PETNR could diffuse.[17] At specific time points, the buffer was removed, replaced, and its absorbance was measured at 464 nm using a PerkinElmer Lambda 25 UV–vis spectrophotometer. Each absorbance was subsequently compared with a calibration graph to determine the concentration of PETNR to give a cumulative release profile over time, minimizing error.

### 2.5. Biotransformation Assay

Determining the quantity of product formed from PETNR in solution (0.2–84 × 10^−6^ m) was undertaken in 1 mL of pH 7 buffer (50 × 10^−3^ m KH_2_PO_4_/K_2_HPO_4_) containing 5 × 10^−3^ m ketoisophorone (added as a concentrated stock solution in ethanol), 10 × 10^−6^ m NADP^+^, 10 units of glucose-6-phosphate dehydrogenase, and 15 × 10^−3^ m glucose-6-phosphate. The reactions were shaken at 37 °C at 130 rpm for 24 h followed by termination of the reaction by extraction with ethyl acetate (0.9 mL) containing 0.5% of limonene as an internal standard. This was subsequently dried using anhydrous MgSO_4_. The extracts were analyzed by gass chromatography (GC) to determine the % yield, % conversion, and enantiomeric excess as described previously.[23] Reactions using the hydrogel samples were performed by placing a 1 mL reaction layer (same as previously stated) on the top of hydrogel and leaving to incubate at 37 °C for 24 h. The reaction layer was subsequently removed, and the reaction was stopped as given above. An additional ethyl acetate extraction was performed over 24 h for the hydrogel to ensure complete removal of any left-over reactant and product.

## 3. Results and Discussion

The peptide used for the fabricationo of the hydrogel is VKVKVEVK, where V, K, and E are valine, lysine, and glutamic acid, respectively. This peptide has been shown previously to self-assemble under physiological conditions into a transparent, β-sheet-rich fibrillar hydrogel at low concentrations.[17] The enzyme selected is PETNR, a member of the Old Yellow Enzyme family of “ene” reductases. These enzymes catalyze the asymmetric reduction of a variety of industrially relevant activated α,β-unsaturated alkenes, including enones, enals, maleimides, and nitro­alkenes.[2,23,31–43]

**Scheme 2:**
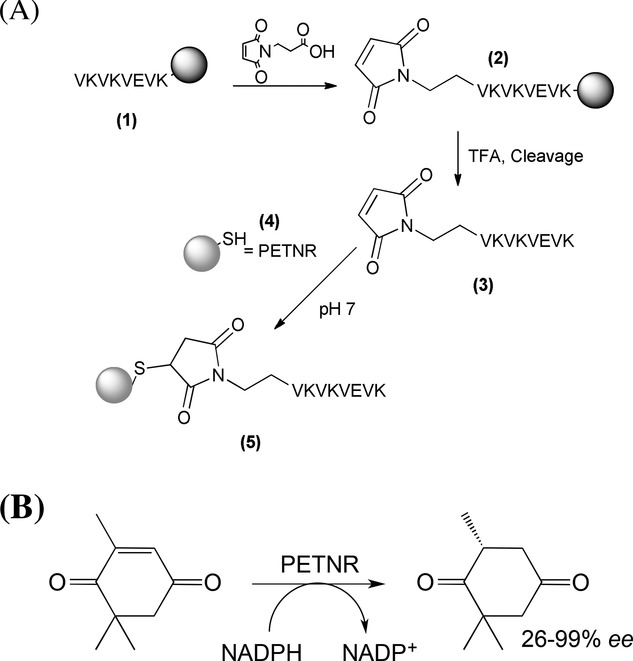
(A) Chemical synthesis of VKVKVEVK–PETNR conjugate (SPep–PETNR) and (B) reduction of ketoisophorone substrate to levodione catalyzed by PETNR.

The chemically coupled VKVKVEVK–PETNR conjugate (SPep–PETNR) was synthesized using a three-step process, as outlined in Scheme 2A. Firstly, VKVKVEVK **(1)** was prepared using standard solid-phase peptide synthesis protocols, as described previously.[17,20,44] A maleoyl linker was attached using standard amino acid coupling conditions, while the peptide was still protected and attached to the solid support **(2)**. Second, the resulting maleimide-functionalized peptide was cleaved from the solid support **(3)** using TFA, and then conjugated to PETNR **(4)** via Michael addition from the native cysteine residue (C222) on the enzyme to the maleimide-end-functionalized peptide. The conjugate **(5)** was analyzed by gel electrophoresis, which showed the conjugate (≈41.5 kDa) had a higher mass than pure PETNR (i.e. > 40.5 kDa) and also higher than the two 40 kDa marker proteins. Matrix-assisted laser light desorption ionization (MALDI) mass spectrometry (Figure S1, Supporting Information) revealed a mass peak at 41.5 kDa, which represents the conjugate. Results from both techniques suggest that the conjugation was successful, indicating that this chemical conjugation is a simple and versatile route that could be used to attach any enzyme containing an exposed cysteine group to any peptide.

The genetically expressed conjugate (CPep–PETNR) was prepared via site-directed mutagenesis of PETNR. The octapeptide VKVKVEVK was incorporated into the C-terminus of an existing highly expressing PETNR construct by PCR, using a modification of the overlapping complementary tails method in two stages.[45] The recombinant enzyme was expressed and purified natively to yield VKVKVEVK-tagged PETNR. Details of the preparation methods are given in the Supporting Information.

Three sets of hydrogels were prepared with VKVKVEVK peptide, containing the following PETNR preparations: 1) free enzyme, 2) SPep–PETNR and 3) CPep–PETNR. In each case 40 mg mL^−1^ of pure peptide was used, as a strong, robust gel is known to form at this concentration at pH 7. The concentrations of PETNR were kept constant at 84 × 10^−6^ m. The phase behavior of each sample was monitored, where a gel is defined when it is self-supporting upon inversion of the test tube. When the system flows, it is defined as a liquid. All samples formed transparent, self-supporting hydrogels; the yellow color indicating the presence of PETNR (an example is given in Scheme 1B). TEM images of the pure VKVKVEVK peptide and the Cpep–PETNR containing system are provided in the Figure S3 (Supporting Information) and reveal the inclusion of the peptide–enzyme component has no impact on fiber formation; both form elongated, flexible fibers of ≈3 nm diameter, which is in excellent agreement with the theoretical length of the fully stretched peptide (2.8 nm), as calculated using molecular modeling in Hyperchem 7.5. Interestingly, all samples remained self-supporting for up to 12 months (longest time explored thus far) suggesting that there was no breakdown in the hydrogel network structure over this time. As the gel preparation method was optimized, it was noted that precipitation occurred instantaneously, and in some cases, macroscopic phase separation occurred, in samples containing both peptide and conjugate when they were subjected to pH values below 6.5 and above 7. This highlights the narrow window under which the enzyme is stable in the presence of large quantities of VKVKVEVK peptide.

**Figure 1:**
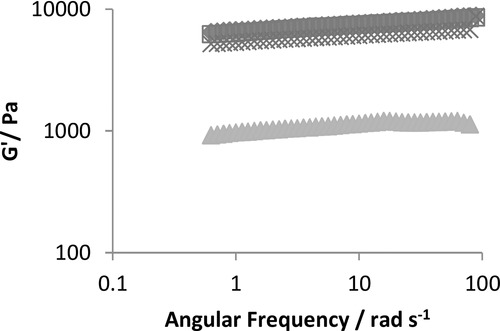
Comparison of the elastic modulus (*G*′) for the pure peptide (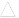), peptide mixed with pure PETNR (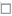) peptide with SPep–PETNR (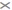) and peptide with CPep–PETNR (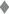). In each case, the concentration of peptide and PETNR was 40 mg mL^−1^ and 84 × 10^−6^ M, respectively.

To confirm the formation of hydrogels, and also to explore the impact of physically incorporating and immobilizing the enzyme within the peptide fibers, the elastic (*G*′) and loss (*G*′′) moduli of all samples were determined using oscillatory rheology. Figure 1A shows the *G*′ obtained for the pure peptide and peptide doped with either, simple PETNR, SPep–PETNR, or CPep–PETNR as a function of radial frequency (1–100 rad s^−1^). In each case, the *G*′ values were at least an order of magnitude higher than the *G*′′, which is indicative of a gelled sample.[46–48] In addition, there was only a weak dependence on the frequency of oscillation in each case, which is also suggestive of a macroscopically viscoelastic solid sample. The value of *G*′ for the pure peptide (≈1000 Pa) is commensurate with our previous work with these octapeptide systems.[17,49] The *G*′ for all samples containing PETNR were significantly higher with values clustering at circa 9000 Pa. Interestingly, this increase in *G*′ is higher than expected; only a slight increase was anticipated due to the presence of 84 × 10^−6^ m of conjugate, which relates to an extra ≈0.09 mg mL^−1^ (0.026%) of peptide contribution from the conjugate. Moreover, the hydrogel containing free enzyme has a similar increase in elastic properties as gels containing the peptide–enzyme conjugate. Therefore, the enzyme must be increased the resistance of the network to shear, presumably by increasing fiber–fiber interactions.

**Figure 2:**
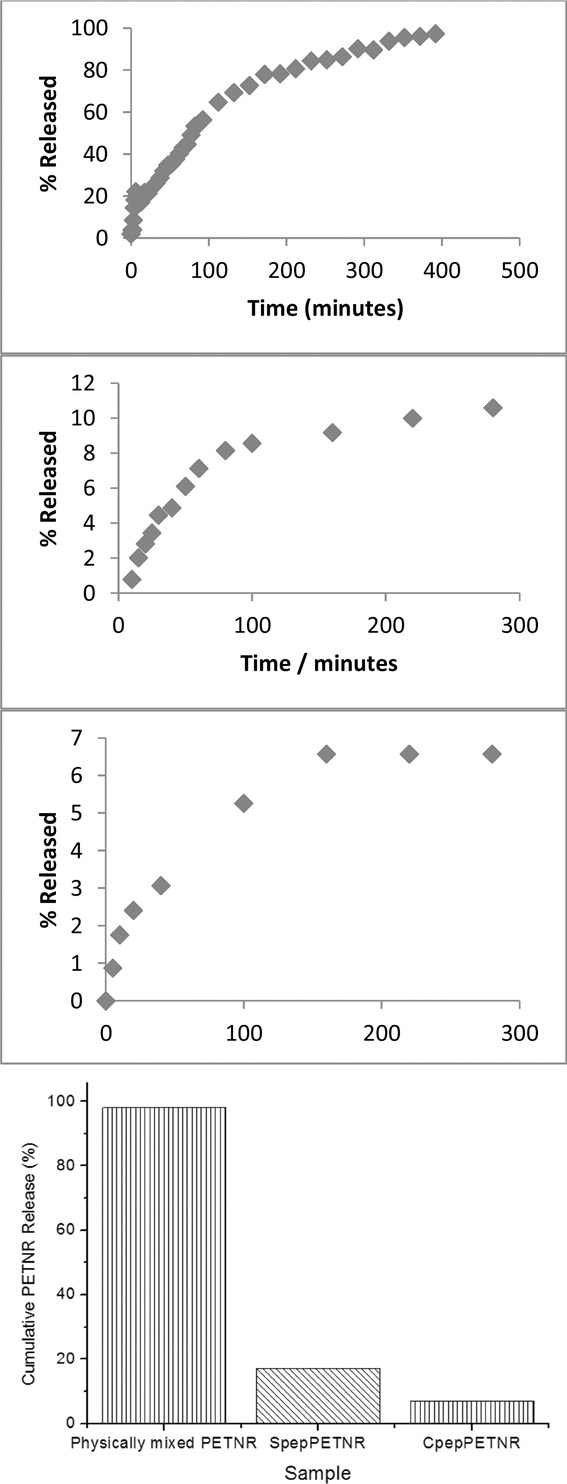
Percentage release of PETNR from 1 mL of (A) physically mixed PETNR as a function of time, (B) SPep–PETNR as a function of time, (C) CPep–PETNR as a function of time, and (D) the cumulative% for the three different peptide hydrogels, into 1 mL of PBS buffer.

To explore whether the peptide component from both conjugates is indeed incorporating within the self-assembled peptide fibers, the quantity of any PETNR released into 1 mL of PBS buffer from 1 mL of each hydrogel sample over time was determined using UV–vis spectro­scopy. Figure 2 shows the results for each of the different hydrogels as a function of time and also a comparison of the cumulative percentages released at equilibrium. It was hypothesized that the enzyme would be immobilized on the nanofiber surface, and hence hinder any diffusion from the hydrogel into the buffer solution over time. It is clear from these data that 98% of the noncovalently linked pure PETNR diffused out of the hydrogel into the buffer solution, and an equilibrium was reached after ≈390 min. Since this is essentially all of the PETNR, it was concluded that its incorporation within the peptide network is thermodynamically unfavorable. This behavior is similar to a recent study by Appel et al.[50] where they found >99% of lysozyme or bovine serum albumin that was physically mixed within a polymer hydrogel was released over a time period of 15–160 d. Such behavior demonstrates that simple noncovalent entrapment of an enzyme within a hydrogel does not significantly enhance stability, or the necessary long-term entrapment required for industrial biocatalytic applications. In the case of hydrogels containing PETNR that was chemically conjugated to the peptide, the release of PETNR was reduced to ≈10%, with an equilibrium reached after ≈210 min. This reduction is most likely due to the peptide component present in the conjugate incorporating within the peptide fibers, thus tethering PETNR to the fiber surface. Such behavior is commensurate with our previous work[44,51] where peptide–polymer conjugates were incorporated homogeneously throughout a pure peptide matrix. Nearly a tenth of all PETNR present was released, however, and could represent a small quantity of conjugate that had not been incorporated into the network, hence it was free to diffuse into the buffer. It could also represent any unconjugated PETNR. The release of PETNR from the hydrogel doped with CPep–PETNR was only ≈7%, with a ≈150 min equilibration time. This again suggests the majority of conjugate is incorporating within the peptide network. These data also indicate that genetic modification is a slightly cleaner route for the preparation of peptide–enzyme conjugates, as no enzyme will be formed via this route without the peptide tag. Interestingly, all release profiles have constant half-lives (*t*_1/2_), and therefore follow first-order release kinetics, suggesting that the only variable between all three samples is the quantity of enzyme available to diffuse into the buffer layer.

To further characterize the immobilized enzyme, we performed biotransformation reactions to assess whether the immobilized PETNR retained activity. PETNR catalyzes the NADPH-dependent double bond reduction of activated alkenes, and has a high specificity for ketoisophorone (Scheme 2B).[23] These results were compared to a control sample of PETNR (no peptide) and a bare hydrogel (no PETNR). All experiments were undertaken within hydrogels where any free enzyme had been removed via diffusion into PBS buffer. The extent of the reactions (% yields of levodione) and product enantiomeric purity were assessed by GC, and the average of at least three separate samples are given in Table1. In all cases, the ketoisophorone was fully consumed, and yields of primarily (*R*)-levodione were constant at ≈86 ± 2%, except for the SPep–PETNR sample in solution which was 71 ± 2% (Table1). The enantiopurities of (*R*)-levodione were greater than 93 ± 1% under all conditions, and were highest for the PETNR solution samples. It is clear, therefore, that enzyme activity and product enantiopreference are retained and improved after enzyme immobilization within the hydrogel compared with previous work[23] where ketoisophorone reduction often gave low optical purity. This demonstrates that the immobilized PETNR has been dramatically improved for industrial biotransformations in comparison to free enzyme. To check that the biotransformation occurred in the bulk of the gel and not simply at the interface, the catalytic rate was determined for 1 mL of gel using vials of different width. No difference was observed confirming that the catalysis occurs within the bulk of the gel sample. We also assessed the thermal stability of free versus immobilized PETNR (a free PETNR solution and CPep–PETNR containing hydrogel) by heating the samples at 90 °C, followed by activity determination at 37 °C. After 10 min, the free enzyme in solution sample precipitated, rendering it inactive. Surprisingly, the CPep–PETNR hydrogel sample retained almost all its activity after being heated overnight at 90 °C, indicating that the environment of the peptide hydrogel is stabilizing the enzyme. Most interestingly, these experiments were repeated on the same samples every month up to a current maximum period of 12 months, and in each case a >99% conversion was observed and the enantiomeric selectivity of the product retained. To determine whether there is any loss of enzyme activity over time, PETNR-functionalized hydrogels were prepared using specifically lower concentrations of enzyme (0.42 × 10^−6^ m) so the standardized biotransformation assays did not give 100% conversion, i.e., the enzyme was not in excess. At initial times, the CPep–PETNR and SPep–PETNR containing hydrogels gave 56 ± 2% and 79 ± 2% conversion of the substrate, respectively. After 12 months storage at room temperature, these conversions were retained. Moreover, the rheological properties were also determined over time and no difference was observed up to the 12 months explored here, confirming the stability of the hydrogels. For similar concentrations of CPep–PETNR and SPep–PETNR in solution, there was no conversion of the substrate after 2 months storage at room temperature. This demonstrates that PETNR retains activity over an extended period of time when incorporated and immobilized within a hydrogel matrix.

**Table 1:** Substrate conversion, yield, and enantiomeric excess of the R-enantiomer from the reductive biotransformation (Scheme 1B). In each case, the peptide was present at 40 mg mL^−1^

Sample	Conversion [%]	Yield [%]	Preferred enantiomer	ee [%]
Bare hydrogel	0	0	–	–
Spep–PETNR (84 × 10^−6^ m) hydrogel	>99	86	R	95
Cpep–PETNR (84 × 10^−6^ m) hydrogel	>99	86	R	93
Spep–PETNR soln. (2 × 10^−6^ m)	97	71	R	>99
Cpep–PETNR soln. (2 × 10^−6^ m)	>99	86	R	>99
PETNR solution, literature[23,42]	>99	80–95	R	26–95
Cpep–PETNR hydrogel (84 × 10^−6^ m)a)	>99	84	–	–
Cpep–PETNR (0.42 × 10^−6^ m) hydrogel	56	5	–	–
Cpep–PETNR (0.42 × 10^−6^ m) hydrogelb)	49	11	–	–
Spep–PETNR (0.42 × 10^−6^ m) hydrogel	79	29	–	–
Spep–PETNR (0.42 × 10^−6^ m) hydrogelb)	71	21	–	–

Peptide–enzyme conjugates are incorporated within self-assembling peptide fibrillar hydrogels to immobilize the enzyme on the surface of the fibers. Once here, they are capable of catalyzing chemical transformations with increased stability and robustness to storage time and temperature.

a) After incubation overnight at 90 °C;

b) After incubation at room temperature for 12 months.

## 4. Conclusions

We have described two different routes to the preparation of peptide–enzyme conjugates for peptide hydrogel incorporation, using the self-assembling octapeptide VKVKVEVK and the biocatalyst PETNR. In the first method, we synthetically coupled VKVKVEVK to the solvent exposed cysteine (C222) of PETNR using well-established click chemistry. The second method involved genetically tagging PETNR with the peptide sequence at the C-terminus of the protein. Both peptide–enzyme conjugates were shown to be embedded throughout the peptide fibrillar network. This demonstrated a functionalized hydrogel where the matrix elasticity and extent of functionalization could be controlled simply by varying the ratio of conjugate and pure peptide. Moreover, we have shown that immobilization of a biocatalyst within a peptide network not only leads to a retention of its activity and product enantioselectivity, but also a significant increase in its longevity, stability, and robustness to time and temperature. This potentially allows reactions to be performed at temperatures outside the usual narrow range where the native enzyme would normally be active. We expect that this novel material technology will open up the possibility of incorporating multiple catalysts within one network, simply via physically mixing different components, and/or the flow of reaction media between different functional hydrogels. Both would allow biocatalytic, cascading chemical transformations for the production of high value, fine chemicals.
