# Supplementary material for: Nanofibrillar Peptide Hydrogels for the Immobilization of Biocatalysts for Chemical Transformations
Source: Macromol Rapid Commun. 2014 Mar 7;35(9):868–74. doi: 10.1002/marc.201400027 (PMC4316184; doi:10.1002/marc.201400027)
Supplement: Supplementary file 1 [file marc0035-0868-sd1.pdf]

Copyright WILEY-VCH Verlag GmbH & Co. KGaA, 69469 Weinheim, Germany, 2014.

Macromolecular Journals

Supporting Information

for *Macromol. Rapid Commun.*, DOI: 10.1002/marc.201400027

Nanofibrillar Peptide Hydrogels for the Immobilization of Biocatalysts for Chemical Transformations

Christopher Hickling, Helen S. Toogood, Alberto Saiani, Nigel S. Scrutton, Aline F. Miller*

Supporting Information

Nanofibrillar peptide hydrogels for the immobilization of biocatalysts for chemical transformations

*C. Hickling, H.S. Toogood, A. Saiani, N.S. Scrutton and A.F. Miller**

Material synthesis: The maleoyl linker (3-(2,5-dioxo-2,5-dihydro-1H-pyrrol-1-yl)propanoic acid) was synthesised from maleic anhydride (Fischer) and β-alanine (Sigma Aldrich) using the de Figueiredo protocol using ethanol as the recrystallization solvent.[52] β-alanine was chosen to minimise hydrophobicity and side reactions. ^{1}H NMR spectrum (Bruker 400 MHz spectrometer at 252 scans) of the linker agreed with previously published results: (400 MHz, $CDCl_3$), δ = 6.72 (s, 2H), 3.83 (t, J = 7.2 Hz, 2H), 2.70 (t, J = 7.2 Hz, 2H). The maleimide functionalized VKVKVEVK peptide was prepared using standard solid phase peptide synthesis (SPPS) using a Discover CEM microwave synthesiser as described elsewhere.[17] Each activation and deprotection step was verified using the Kaiser test.[53] Addition of the maleoyl linker was achieved without microwave irradiation at room temperature for 6 hrs. Subsequent cleavage from the Wang resin (Merck) was done using 95% trifluoroacetic acid (TFA) (Sigma Aldrich) and 5% anisole (Sigma Aldrich) before the product was verified by mass spectrometry (m/z = 1079 g mol^{-1}), and its purity determined using HPLC (~85%). Coupling of the functionalised peptide to PETNR-His$_8$ was done by dissolving 10 mg of the peptide in 1 ml of pH 7 ammonium acetate (NH_4OAc) buffer solution (10 mM EDTA) (both Fischer), and adding 800 µL of PETNR-His$_8$ solution (17.05 mg ml^{-1} in 50mM TRIS pH 7.0) The reaction mixture was cooled and stirred in an ice bath (~4°C) for 2 hrs. Conjugation was confirmed via gel electrophoresis and matrix assisted laser desorption ionisation-time of flight (MALDI-TOF) (**Figure S1**).

*Recombinant conjugate synthesis: C-Peptide tag incorporation***.** A C-terminal octapeptide tag (VKVKVEVK) was incorporated into an existing highly expressing PETNR construct (pONR1; pBluescript SK^{+})[30] by PCR, using a modification of the overlapping complementary tails method in two stages[42] (**Figure S2**). This method is based on the QuikChange whole plasmid synthesis protocol (Stratagene). In the first stage, the forward and reverse primers (PepIns4F and PepIns4R) contain the DNA sequence of the last and first 12 bases of the tag, respectively. This generates a blunt ended, nicked PCR product containing the full sequence of the tag. To improve transformation efficiency, a second stage was incorporated using primers PepIns8F and PepIns8R, each of which contains the full peptide sequence. This generates a PCR product with a 24 base overhang. To simplify the protocol, the PCR reaction contained both sets of forward and reverse primers, with the second stage pair in 4-fold excess of the first stage pair. The primers used were as follows (Eurofins MWG Operon): PepIns4F 5'-GTGGAAGTGAAATAATCCCGCTTTGTACATTGATAGCG-3'; PepIns4R 5'-TTTCAC TTTCACCAGTGAAGGGTAGTCGGTATAACCTTC-3'; PepIns8F 5'-GTGAAAGTGAA AGTGGAAGTGAAATAATCCCGCTTTGTAC-3' and PepIns8R 5'-TTTCACTTCCAC TTTCACTTTCACCAGTGAAGGGTAGTC-3'.

PCR products were visualised using 0.6% agarose gel electrophoresis in TAE buffer (40 mM Tris pH 8.5 containing 1.14 mL glacial acetic acid and 4 mM EDTA) for 30 minutes at 110 V. DNA fragments of the expected size were gel purified according to the manufacturers protocol (Qiagen). Constructs were transformed into the *E. coli* strain JM109 (Promega), according to the manufacturer’s protocol, and incubated on Luria broth (LB) agar containing ampicillin (100 μgmL^{-1}) for 24 hours at 37°C. Insertion clones were identified by DNA sequencing (Eurofins MWG Operon) of plasmid DNA isolated from randomly picked transformation colonies. Glycerol stocks of the positive clone were generated by combining an

equal volume of overnight culture and sterile 2 × glycerol stock buffer (phosphate buffered saline:glycerol 80:20).

Recombinant conjugate synthesis: PETNR production: All PETNR constructs (C-terminally His_8-tagged and VKVKVEVK–tagged) were produced by inoculating 12 × 1 L Teriffic broth (Formedium), containing buffer salts (17 mM KH_2PO_4 and 72 mM K_2HPO_4), 0.2% glycerol and 100 μg mL^{-1} ampicillin, with an overnight starter culture grown in Luria broth (Formedium) containing 100 μg mL^{-1} ampicillin. The cultures were shaken at 37ºC (200 rpm) for several hours until the absorbance at 600 nm ~ 0.5-0.8. PETNR expression was induced by the addition of 0.1 mM IPTG, and a further incubation of 16 hours at 30ºC. Cultures were harvested by centrifugation at 1000 *g* for 10 minutes at 4ºC. Cell pellet was frozen in liquid nitrogen, and stored at −80ºC.

Recombinant conjugate synthesis: PETNR purification. C-terminally His_8-tagged PETNR used for the chemically attached peptide method was purified according to previously published methods.[42] Non-His-tagged PETNR cells were resuspended in lysis buffer (50 mM KH_2PO_4/K_2HPO_4 pH 6.5 containing Complete EDTA-free protease inhibitor tablets (Roche), 1 mg FMN (Sigma), 0.01 mg mL^{-1} lysozyme and DNAse (Sigma)) and stirred for 20 minutes at 4ºC. Cells were lysed by sonication in an ice water bath, followed by extract clarification by centrifugation (54,000 *g*) for 1 hour at 4ºC. The supernatant was dialysed against 3 × 5 L of equilibration buffer 1 (10 mM KH_2PO_4/K_2HPO_4 pH 6.5) at 4°C. The enzyme was filtered (0.2 μm) and applied to a Mimetic orange column (Prometic Biosciences). PETNR eluted isocratically in elution buffer, and dialysed against 3 × 5 L of equilibration buffer 2 (50 mM Tris pH 8.0). The enzyme was applied to a Q-Sepharose column (GE Healthcare), and washed with equilibration buffer 2. Protein was eluted with a gradient to 100% elution buffer (50 mM Tris pH 8.0 containing 1M NaCl). Purified protein fractions were pooled and dialysed against 3 × 5 L of

storage buffer (10 mM Tris pH 7.0), and concentrated by ultrafiltration on a PM30 membrane. PETNR presence and purity during each purification stage was detected by SDS PAGE. The concentration of total and active protein was determined using the Bradford[54] and extinction coefficient methods,[31] respectively (ε_{464nm} = 11,300 M^{-1} cm^{-1}).

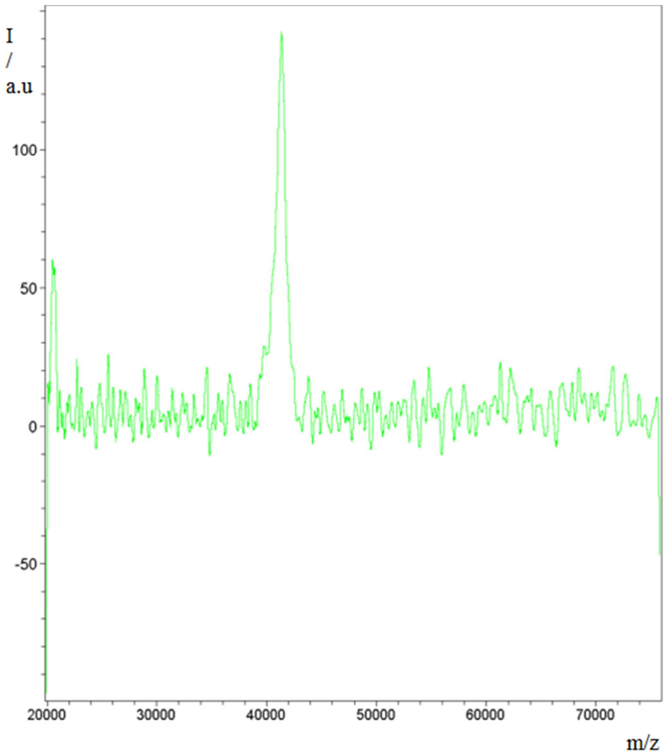

Figure S1. MALDI spectrum of a chemically coupled conjugate between mal-VKVKVEVK and PETNR-His_8, with a distinct peak at ~41.5 m/z.

The resultant MALDI spectrum yields a peak at ~41500 m/z, which corresponds to the expected mass of the conjugate between PETNR-His_8 and mal-VKVKVEVK, when a single charge is present. This provides evidence towards maleoyl linker being a suitable method of conjugating VKVKVEVK to PETNR-His_8.

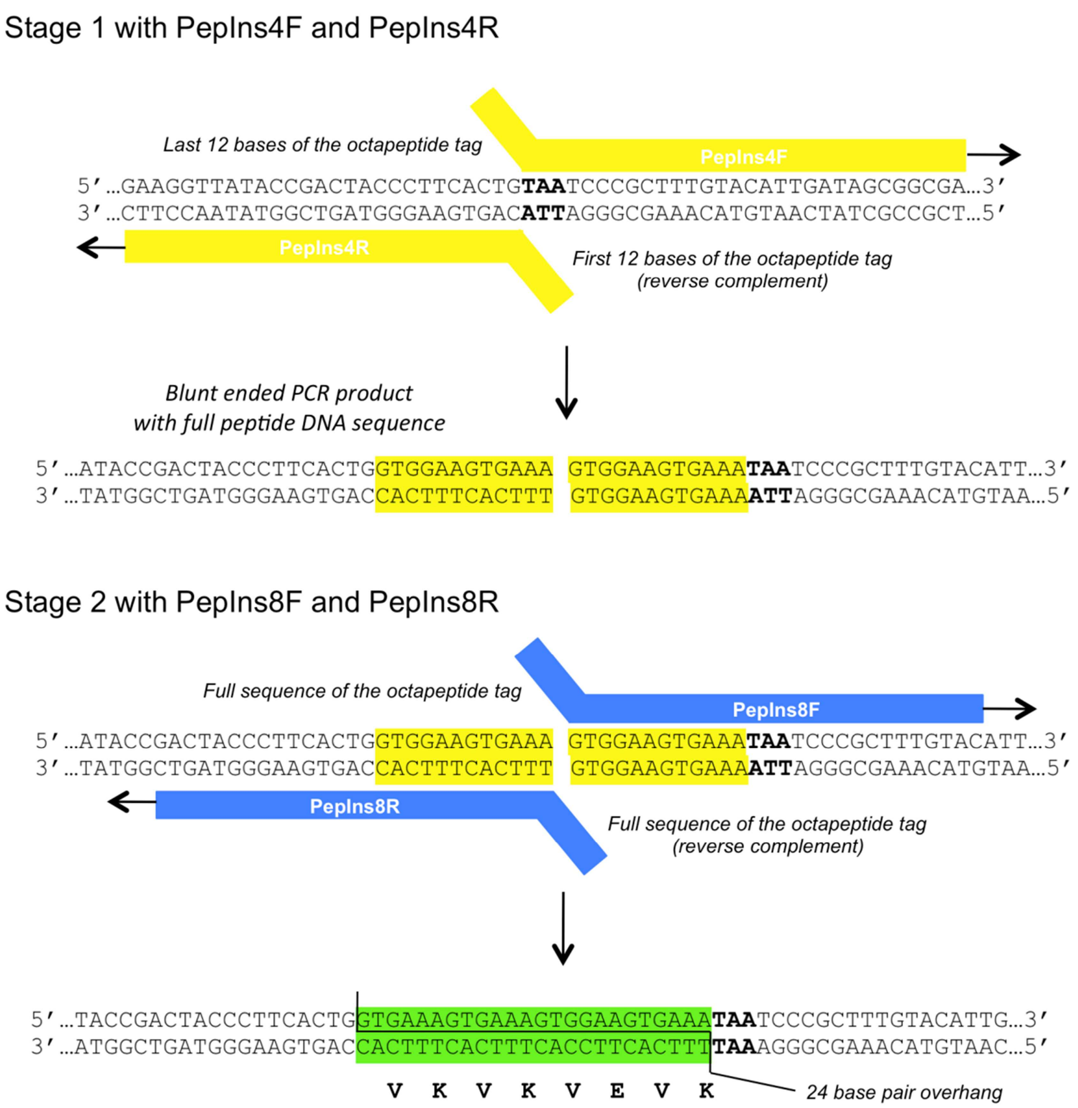

Figure S2. Schematic representation of the modified overlapping complementary tails method of octapeptide tag insertion into PETNR. Both stages were performed in the same PCR reaction due to the presence of all 4 primers.

(A)
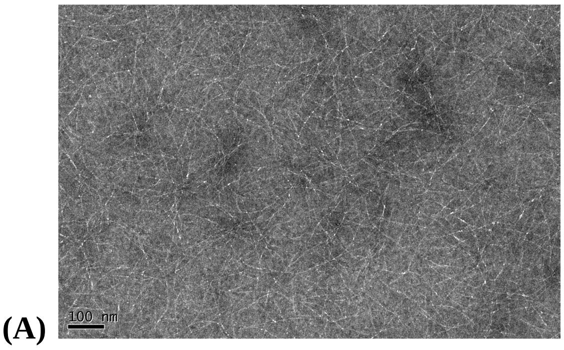

(B)
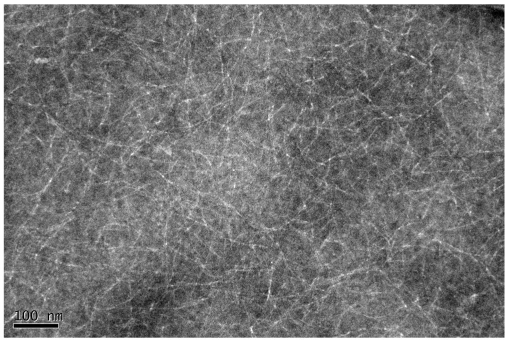

Figure S3. TEM micrographs of (A) pure VKVKVEVK peptide hydrogel and (B) VKVKVEVK with Cpep-PETNR. The scale bar in each case represents 100 nm.

Copyright WILEY-VCH Verlag GmbH & Co. KGaA, 69469 Weinheim, Germany, 2013.

Supporting Information

for *Macromol. Chem. Phys*, DOI: 10.1002/macp.2013#####

for *Macromol. Rapid Commun.*, DOI: 10.1002/marc.2013#####

for *Macromol. Biosci.*, DOI: 10.1002/mabi.2013#####

for *Macromol. React. Eng.*, DOI: 10.1002/mren.2013#####

for *Macromol. Theory Simul..*, DOI: 10.1002/mats.2013#####

for *Macromol. Mater. Eng.*, DOI: 10.1002/mame.2013#####
